# Unveiling the impact of CDK8 on tumor progression: mechanisms and therapeutic strategies

**DOI:** 10.3389/fphar.2024.1386929

**Published:** 2024-03-28

**Authors:** Xiaomin Yin, Zhilong He, Kun Chen, Kai Ouyang, Changxuan Yang, Jianjun Li, Hailin Tang, Manbo Cai

**Affiliations:** ^1^ Department of Radiotherapy, The First Affiliated Hospital, Hengyang Medical School, University of South China, Hengyang, Hunan, China; ^2^ Department of Urological Surgical, The Second Affiliated Hospital, Hengyang Medical School, University of South China, Hengyang, China; ^3^ State Key Laboratory of Oncology in South China, Guangdong Provincial Clinical Research Center for Cancer, Sun Yat-Sen University Cancer Center, Guangzhou, China

**Keywords:** tumorigenesis, CDK8, immune, cell cycle, invasion, energy metabolism, DNA damage

## Abstract

CDK8 is an important member of the cyclin-dependent kinase family associated with transcription and acts as a key “molecular switch” in the Mediator complex. CDK8 regulates gene expression by phosphorylating transcription factors and can control the transcription process through Mediator complex. Previous studies confirmed that CDK8 is an important oncogenic factor, making it a potential tumor biomarker and a promising target for tumor therapy. However, CDK8 has also been confirmed to be a tumor suppressor, indicating that it not only promotes the development of tumors but may also be involved in tumor suppression. Therefore, the dual role of CDK8 in the process of tumor development is worth further exploration and summary. This comprehensive review delves into the intricate involvement of CDK8 in transcription-related processes, as well as its role in signaling pathways related to tumorigenesis, with a focus on its critical part in driving cancer progression.

## 1 Introduction

Transcription is a fundamental cellular process that transforms genetic information into an inducible molecular phenotype, and is also the initial stage in the expression of cellular genetic information. This essential, preserved, and intricately controlled biological mechanism is carefully coordinated to guarantee accurate synchronization between the genetic blueprint and cellular requirements. Transcription dysregulation has been linked to a range of human illnesses such as cancer. Transcriptional regulation is a multifaceted process that entails multiple transcription factors, including activators and inhibitors, coactivators and coinhibitors, RNA pol II, and general initiation factors. Mediator is a widely required transcriptional coactivator. The recent discovery that CDK8 is an integral component of the reversible CDK8 submodule within L-Mediator prompted us to investigate its pivotal role in transcriptional processes (Bourbon et al., 2004). The CDK8 protein kinase, consisting of 464 amino acids, frequently exhibits amplification in colorectal cancer (Firestein et al., 2008; He et al., 2011). Moreover, the involvement of CDK8 in the pathogenesis of gastric cancer, breast cancer, prostate cancer, melanoma, and acute myeloid leukemia highlights its potential as a promising therapeutic target for various malignancies (Kapoor et al., 2010; Broude et al., 2015; Nitulescu et al., 2017; Offermann et al., 2022; Xu et al., 2023). However, CDK8 exerts an anticancer role in endometrial cancer in addition to its role in tumor growth, implying that CDK8 has a different function in the growth of cancer (Gu et al., 2013). Indeed, CDK8 plays an intricate role in malignancies. In this review, we first elucidate the role of CDK8 in the transcription process, and then explore its biological functions in cancer, including the cycle change, invasion and migration, tumor metabolism, DNA damage and immune.

## 2 Biochemistry of CDK8

### 2.1 CDK8 kinase module (CKM)—a special mediator complex

The transcription of proteins in eukaryotes necessitates the involvement of a holoenzyme known as RNA polymerase II (Pol II). This intricate enzyme complex, comprising 12 subunits, is responsible for transcribing DNA into mRNA (Lee and Young, 2000; Shilatifard et al., 2003). Multiple transcription factors (TFs, classified as transcriptional activators and inhibitors) must work together to regulate transcription via Pol II. TFs are DNA-binding proteins that can bind to target sequences in transcriptional regulatory regions and regulate the assembly and activity of transcriptional mechanisms by acting directly on protein interactions or by exerting an effect on chromatin (structural remodeling, histone modification, etc.). These functions of transcription factors are further regulated by transcription coregulators (Lambert et al., 2018).

The Mediator complex, a transcriptional coactivator, functions as an intermediary connecting TFs with RNA pol II by receiving the signal of transcriptional activation carried by these TFs and delivering it to the polymerase. In eukaryotes, the assembly of the Mediator complex, RNA pol II and various TFs constitutes a pre-transcriptional initiation complex (PIC), which is indispensable for initiating transcription (Kornberg, 2005; Taatjes, 2010). The process of transcriptional regulation involves two distinct components of the mediator complex, namely, the CDK8 kinase module and the core mediator complex (Bourbon et al., 2004). A crucial aspect of the regulation of RNA pol II by PIC involves direct (via RNA pol II phosphorylation) or indirect control of RNA pol II activity by distinct kinases. One of the highly conserved functions of these kinases is their key role in the regulation of gene transcription initiation, elongation, and RNA processing by phosphorylating the C-terminal domain (CTD) of RNA pol II. This intricate process necessitates the participation of three distinct members of the cyclin-dependent kinase (CDKs) family, namely, CDK7, CDK8, and CDK9 (Myers et al., 1998; Pinhero et al., 2004; Tsai et al., 2013).

CDKs are serine/threonine protein kinases that play pivotal roles in regulating transcriptional processes and the cell cycle. They can be divided into two categories: (i) CDKs associated with cell cycle progression, such as CDK1, CDK2, CDK4, and CDK6, and (ii) transcription-associated CDKs like CDK7, CDK8, CDK9, CDK12, and CDK13 that orchestrate gene transcription by phosphorylating the CTD of RNA pol II (Malumbres, 2014).

CDK8 is a protein made of 464-amino-acid and binds to cyclin C (CCNC), MED12, and MED13 to form the CDK8 module, where CDK8 is the main functional subunit of this module. As a component of the kinase module, CDK8 binds reversibly to the Mediator complex. Furthermore, CDK8 effectively safeguards cyclin C against proteolytic degradation through a kinase-independent mechanism (but not MED12 or MED13), but its kinase activity may be detrimental to the three parts of mediator-associated CDK modules (cyclin C, MED12, and MED13) (Chen et al., 2023). Nevertheless, cyclin C and MED12 are needed for the activation of CDK8 kinase, while MED13 plays a crucial role in attracting preassembled kinase modules to the core mediator, which, once bound, lead to significant structural and functional alterations within the core mediator (Knuesel et al., 2009a; Knuesel et al., 2009b; Tsai et al., 2013).

Similar to other transcription-related CDKs, CDK8 participates in gene transcription by phosphorylating the CTD domain of RNA pol II with its phosphokinase activity. For instance, CDK8 phosphorylates CTD repeats at Ser2 and Ser5 *in vivo* and *in vitro* (Pinhero et al., 2004; Donner et al., 2010). In addition, CDK8 controls transcription by joining with Mediator complex as the primary functional subunits of the CDK8 module (Knuesel et al., 2009a).

Interestingly, CDK8-mediated kinase exerts positive regulation on genes during the early stage of transcription and switches to negative regulation during the late stage. For instance, treatment with Senexin B, a highly specific CDK8/19 inhibitor, predominantly downregulated gene expression after 3–5 h; however, between 24 h and 15 days, gene expression was primarily upregulated. These findings imply that Mediator kinase functions as a positive regulator of early-response genes (Chen et al., 2023).

### 2.2 CDK8 regulates transcription

#### 2.2.1 CDK8 exerts transcriptional regulation by association with the mediator complex

The process of transcription can be categorized into three phases: initiation, elongation, and termination, and initiation can be further subdivided into three sequential steps. First, the RNAP holoenzyme forms a PIC by binding to the promoter DNA, which acts as a blocking complex. Subsequently, an open complex is formed by the single-stranded DNA surrounding the initiation site. Finally, a “promoter escape” process is used to transition from inception to extension (Nogales et al., 2017).

**FIGURE 1:**
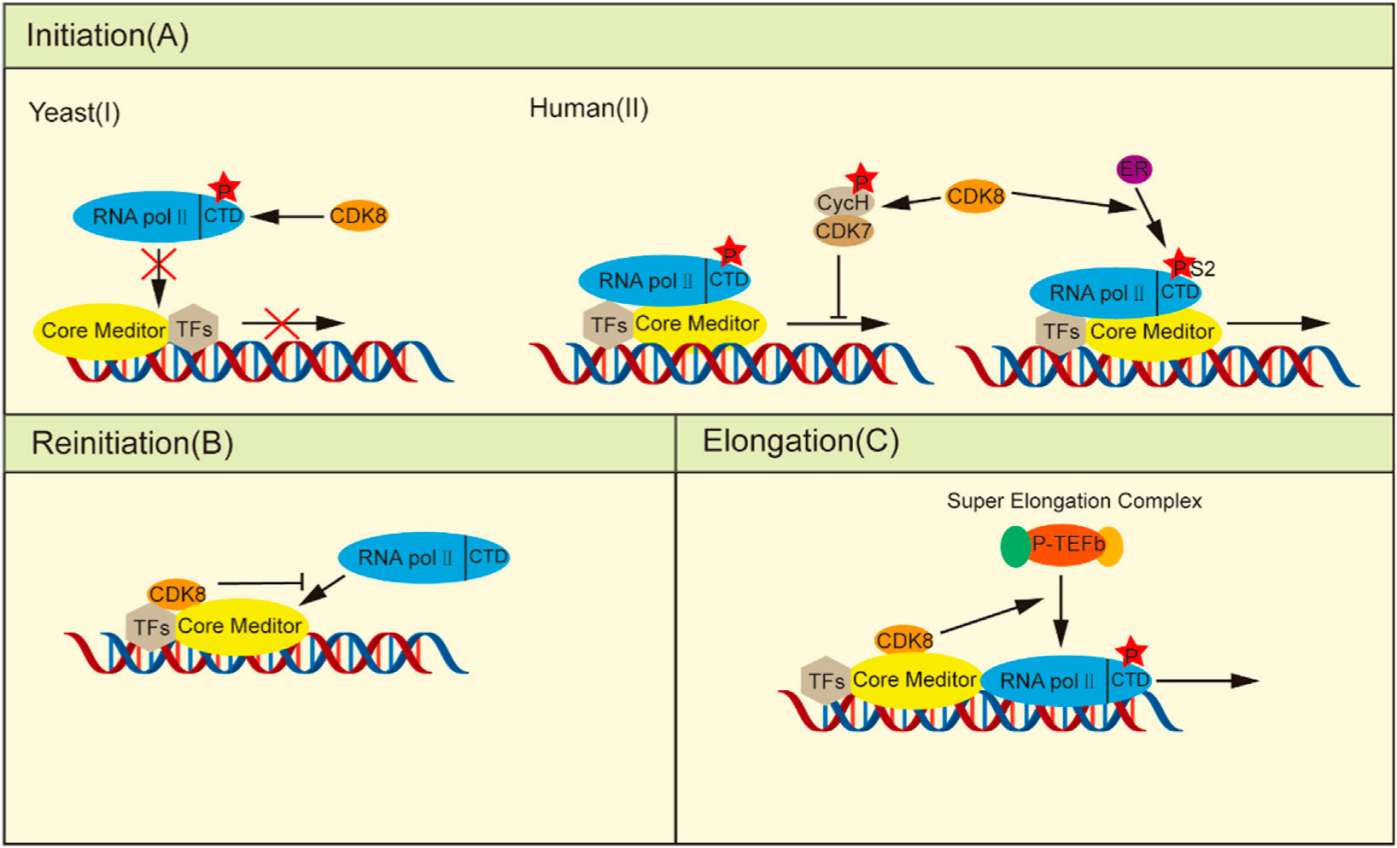
CDK8 regulates transcription. **(A)** In the initiation phase: (I) Yeast CDK8 phosphorylates the RNA pol II CTD, preventing its integration into the PIC and consequently negatively regulating transcription. (II) Human CDK8 transcription has a dual role: on the one hand, phosphorylation of cyclin H, thereby deactivating CDK7 to prevent transcription initiation, can be induced by stimulation of estrogen RNA pol II CTD Ser2 phosphorylation, thus facilitating ER-induced gene transcription. **(B)** Upon the escape of the RNA pol II promoter, the integration of kinase modules into the core plate induces a conformational alteration in the core mediator, thereby impeding its interaction with other RNA pol II molecules. **(C)** In the elongation phase, CDK8 facilitates the recruitment of P-TEFb, promoting transcriptional elongation.

During the initial phase, yeast CDK8 phosphorylates the CTD of RNA pol II *in vitro*, causing its PIC assembly to be blocked and thus negatively regulating transcription (Figure 1A) (Hengartner et al., 1998; Galbraith et al., 2010). Human CDK8 phosphorylates Ser5 and Ser304 of Cyclin H *in vitro*, which brings about loss of CDK7 kinase activity and prevents initiation of transcription (Boeing et al., 2010; Wong et al., 2014; Luo and Horvitz, 2017). However, the depletion of CDK8 in HCT116 cells did not exert any influence on transcription initiation despite the fact that phosphorylation at Ser2 and Ser5 of the RNA pol II CTD was significantly reduced (Donner et al., 2010). Research has shown that CDK8 enhances the estrogen receptor (ER) in breast cancer by stimulating Ser2 phosphorylation of estrogen-induced RNA pol II CTD, thus more effectively assisting in the transcription process of ER-inducible genes, such as *GREB1*、*CXCL12* and *TFF1* (Figure 1A) (McDermott et al., 2017).

Furthermore, CDK8 can efficiently inhibit transcriptional reinitiation. Once initiated, RNA pol II dissociates from the PIC, leaving behind a scaffold complex that facilitates subsequent rounds of transcriptional reinitiation (Yudkovsky et al., 2000). When the kinase module binds to the core mediator, the core mediator changes conformation as a result of the creation of the CDK8-Mediator complex, rendering the change incompatible with the binding of RNA pol II and acting as a molecular correct switch (Taatjes et al., 2002; Knuesel et al., 2009a). Additionally, the competitive inhibition of the binding between the kinase module and RNA pol II to the core mediators is observed (Figure 1B) (Näär et al., 2002; Ebmeier and Taatjes, 2010). Thus, after the initiation of transcription, the formation of the CDK8-Mediator complex is able to rapidly inhibit the reassociation of the second RNA pol II with the promoter, effectively preventing the resumption of transcription.

In addition to its role before and after transcription initiation, CDK8 also enhances the expression of stimulus-specific genes by modulating transcriptional elongation processes. To adapt to hypoxia under certain stress conditions, cells need transcriptional responses provoked by cellular hypoxia-inducing factors (HIFs). Transactivation of hypoxia inducible Factor 1 alpha (HIF1α) -induced genes necessitates the involvement of the CDK8-Mediator complexes to facilitate the activation of HIF1α-targeted gene expression through the recruitment of positive transcription elongation factor-b (P-TEFb) and super elongation complexs (SECs) (Figure 1C) (Galbraith et al., 2013; Perez-Perri et al., 2016). In HCT116 cells, knockdown of CDK8 resulted in a significant decrease in the phosphorylation of CTD of RNA pol II at Ser2 and Ser5, accompanied by impaired transcription of immediate early genes (IEGs) induced by serum stimulation. However, it had no impact on RNA pol II recruitment, except for the recruitment of SEC to IEG gene sites. This is attributed to the indispensable role of CDK8 in facilitating recruitment of the transcriptional elongation regulators bromodomain containing 4 (BRD4) and P-TEFb (Donner et al., 2010). Similarly, another study demonstrated the essential role of the CDK8-Mediator complex in transactivating hypoxia-inducible genes that rely on HIF1α under hypoxic conditions by facilitating recruitment of the SEC to promote releasing paused RNA pol II (Galbraith et al., 2013).

#### 2.2.2 CDK8 regulates transcription by directly phosphorylating TFs

CDK8 is involved in transcriptional regulation by directly affecting TFs activity or triggering ubiquitination degradation through the phosphorylation of TFs. The action of CDK8 in regulating its phosphorylation level and activity is mediated through direct interactions with TFs, independent of the Mediator complex, thereby modulating the signaling pathway. TFs associated with CDK8 phosphorylation include the SMAD, NOTCH, STAT, and SREBP families.

**FIGURE 2:**
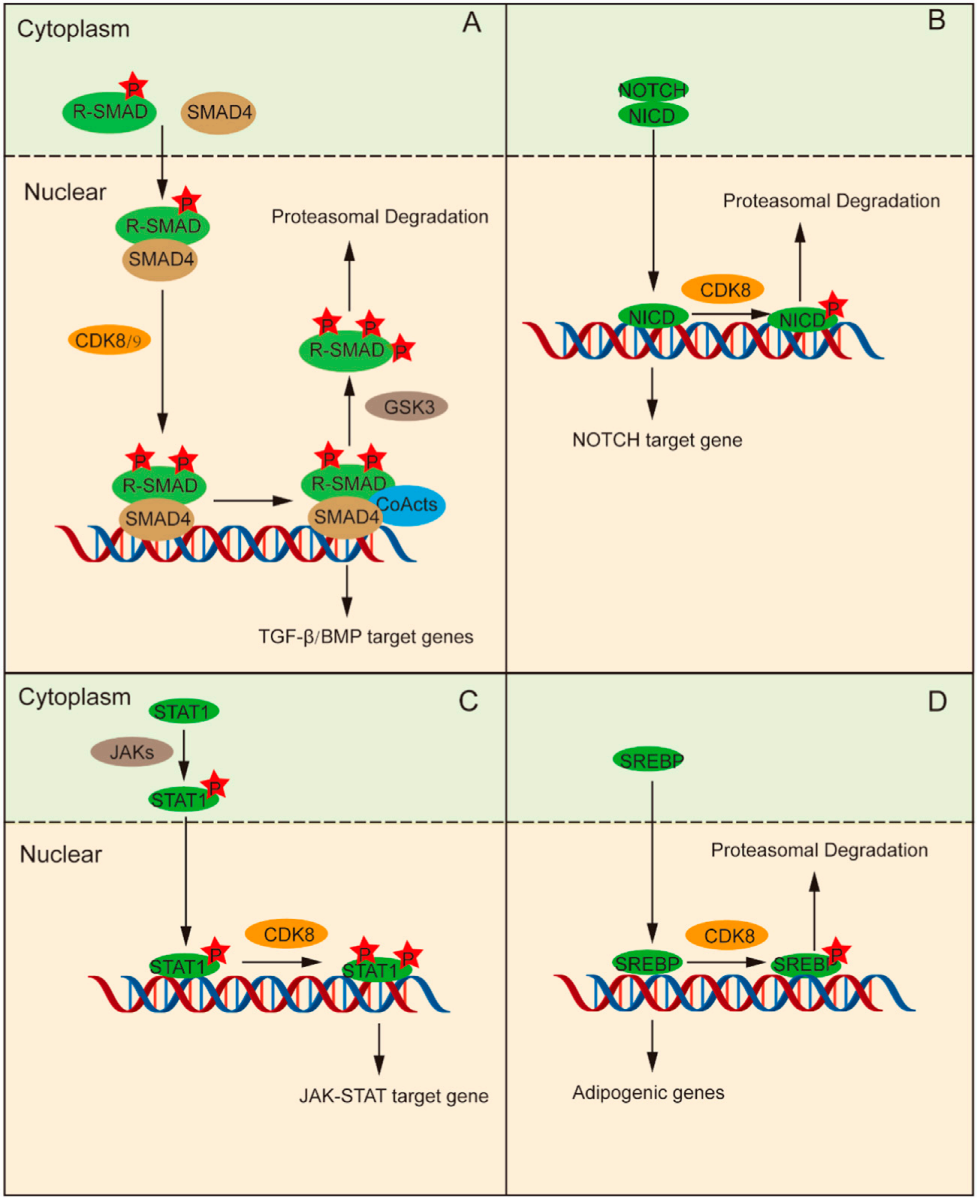
CDK8 regulates transcription by phosphorylating transcription factors directly. **(A)** Phosphorylation of R-Smads and Smad4 assembly occur in the cytoplasm, followed by their transfer to the nucleus. Then, CDK8 is further phosphorylated, which can activate transcription factor interactions to promote the phosphorylation of R-Smads that can be ubiquitinated and degraded. **(B)** NICD enter into the nucleus and stimulates CDK8 to control the function of NICD by phosphorylating NICD, followed by ubiquitination and degradation, thus terminating the NOTCH signaling pathway. **(C)** Tyr701-phosphorylated STAT1 forms a dimer, which is conveyed to nucleus for subsequent phosphorylation of STAT1a by CDK8/CCNC to promote transcription. **(D)** CDK8/CCNC induces increased ubiquitination and protein degradation of SREBP through phosphorylation of SREBP.

The TGF-β signaling pathway plays an pivotal role in tissue and organ morphogenesis, tissue repair, immune supervision, and maintenance of adult homeostasis (Macias et al., 2015). Smad proteins are signal transduction molecules downstream of the TGF-β receptors and are divided into three categories according to their functional differences in TGF-β signal transduction: receptor-regulated Smad (R-Smad), universal Smad (Co-Smad), and suppressed Smad (I-Smad) (Derynck and Zhang, 2003). R-Smads phosphorylated in the C-terminal domain form complexes with Smad4 and undergo nuclear transport, where CDK8/CDK9 further phosphorylates the ligand subregion of R-Smads (Alarcón et al., 2009; Khan and Zhang, 2019). This phosphorylation enhances the counter activation of R-Smads by promoting their interaction with coactivators. Moreover, it can promote Smad protein phosphorylation by the GSK3 protein, which can promote Smad protein-specific E3 ubiquitination ligases SMURF1 and NEDD4L to bind to the Smad protein, and mediate Smad protein ubiquitination and protease degradation, thus repressing the expression of target genes (Figure 2A) (Fuentealba et al., 2007; Gao et al., 2009). Hence, CDK8/CCNC-mediated phosphorylation is implicated in the maintenance of Smad protein levels and augments the transcriptional activity of Smad.

The NOTCH signaling pathway gives full play to a pivotal role in determining cell fate and is indispensable for T cell differentiation, intercellular communication, and glial development (Gaiano and Fishell, 2002; Dontu et al., 2004; Laky and Fowlkes, 2008). The extracellular domain of the NOTCH receptor binds to its ligand and releases the NOTCH intracellular domain (NICD), which enters the nucleus and stimulates the transcription of NOTCH-regulated target genes (Farshbaf et al., 2015). Subsequently, CDK8 phosphorylates NICD in the nucleus, leading to its ubiquitination and degradation, thereby terminating NOTCH pathway activation and ensuring the stability of NICD protein levels as well as regulation of its function (Figure 2B) (Fryer et al., 2004; Popko-Scibor et al., 2011).

The JAK-STAT pathway activated by cytokines plays a crucial role in a variety of important biological processes and is an inflammatory signaling pathway for stress (Levy and Darnell, 2002). Upon stimulation by IFN-γ signaling, JAK phosphorylates STAT1 at Tyr701 leading to the formation of a dimer which subsequently translocates into the nucleus for binding to target genes and promoting their transcriptional activity. C-C motif chemokine ligand 5 (CCL5), for example, is activated by STAT via PTBP2-mediated, induces monocyte/macrophage chemotaxis (Tang et al., 2023). STAT1 indirectly regulates the expression and activity of IRF9 through PTBP2-mediated alternative splicing. STAT1 binds to the promoter region of CCL5, promoting its transcription and thereby influencing the chemotaxis and repolarization of tumor-associated monocytes/macrophages. However, it should be noted that the phosphorylation of STAT1-Ser727 is also essential for achieving complete IFN-γ-dependent gene expression (Varinou et al., 2003; Sadzak et al., 2008). CDK8/CCNC mediates the subsequent phosphorylation of STAT1 at Ser727. Thus, STAT1-dependent transcription of the IFN-γ signal is fully activated (Figure 2C) (Bancerek et al., 2013).

The SREBP family includes key transcription factors that regulate lipid metabolism. SREBP-1C becomes proteolytic in the Golgi apparatus under the stimulation of insulin signaling and is then transported to the nucleus to trigger the transcription of adipogenic genes (Yokoyama et al., 1993). CDK8/CCNC promotes enhanced ubiquitination and proteasomal degradation of SREBP-1C by phosphorylating Thr402 residue on SREBP-1C (Figure 2D) (Yokoyama et al., 1993; Feng et al., 2015).

RAS/MAPK signaling pathway is a crucial regulator of cell growth, and aberrant RAS/MAPK signaling is a common driver of tumorigenesis, with its downstream target ERK typically activated through phosphorylation. Targeted therapy using MEK inhibitors has been approved for clinical use. However, in advanced cancer, using MEK inhibitors as a monotherapy can induce compensatory upregulation of pro-growth gene expression, leading to limited clinical outcomes in many Ras-driven cancers (Eleveld et al., 2015). A study found that in neuroblastoma, the combined use of CDK8 inhibitor BI-1347 and MEK inhibitor trametinib, compared to using trametinib alone, can reduce the phosphorylation levels of ERK. This suggests that BI-1347 may inhibit the transcriptional function of MEK. Gene set analysis revealed that genes and gene sets affected by the upregulation of MEK inhibition and downregulation of CCNC and CDK8 showed an overall downregulation or remained unchanged trend. These results indicate that under MEK inhibition, the kinase activity of CDK8/CCNC is necessary for the compensatory upregulation of growth-promoting gene expression programs, and inhibiting CDK8/CCNC would prevent this transcriptional adaptation (Malone et al., 2023).

## 3 Emerging roles of CDK8 in tumorigenesis

### 3.1 CDK8 controls the cell cycle

The cell cycle is a strictly controlled process that encourages cell division, gene replication, and cell growth. Cells advance from one cell cycle phase to the next by employing the essential cell cycle machinery that works in the nucleus. The cell cycle-specific transcription of cyclins, protein degradation, and a number of signaling channels all work together to strictly regulate their activity in normal cells. However, in malignancies, these elements are frequently out of control, which causes abnormal cyclin activation. The p53 transcriptional network is a well-known tumor suppressor and a crucial route that coordinates cell cycle arrest (Hu et al., 2021). CDK8 functions as a coactivator in the p53 transcriptional program, and enhanced CDK8 binding to p53 target genes is positively linked with transcriptional activity. The p21 gene is one of the targets regulated by CDK8, which is a negative regulator of CDK1 and CDK2 complexes and a key target gene for p53-dependent cell cycle arrest (Donner et al., 2007). By activating p21, p53 inhibits cells from entering the division phase while also arresting cells in the G1 phase (Engeland, 2022). Three subunits of the intermediate CDK module (CDK8, MED12, and cyclin C) are specifically recruited to the p21 promoter to stimulate transcription under conditions of strong p21 transcriptional activation (Donner et al., 2007). The expression of p21 in turn causes further stimulation of CDK8 kinase activity, establishing a positive feedback loop between CDK8 and p21 expression (Porter et al., 2012).

The Wnt/β-catenin signaling pathway is reportedly involved in the abnormal proliferation of colorectal cancer cells (Zhao et al., 2022). Activation of Wnt/β-catenin signaling leads to an increase in β-catenin in the nucleus, which promotes cyclin D gene expression and simultaneously inhibits the expression of p21 and p27, ultimately driving cells into S phase (Szilagyi and Gustafsson, 2013). CDK8 is a key tumor promoter in colorectal cancers. Mediators and CDK8 have been found to be directly connected to the Wnt/β-catenin pathway. Colon cancer cells were unable to proliferate and were arrested in the G0/G1 phase when CDK8 was knocked down (He et al., 2011). In addition, CDK8 further controls p27 levels. CDK8 drives the phosphorylation of p27 at residue T187, providing an initiation site for Skp2 to promote Skp2-mediated ubiquitination and degradation of p27. In Skp2-deficient mice, deletion of p27 reverses G2/M arrest. However, this is not observed in breast cancer cells, possibly as a result of the various genetic abnormalities present in them, including p53 mutations, some of which may influence the effects of CDK8, mH2A1, and Skp2 on G2/M arrest and polyploidy (Xu D. et al., 2015).

**FIGURE 3:**
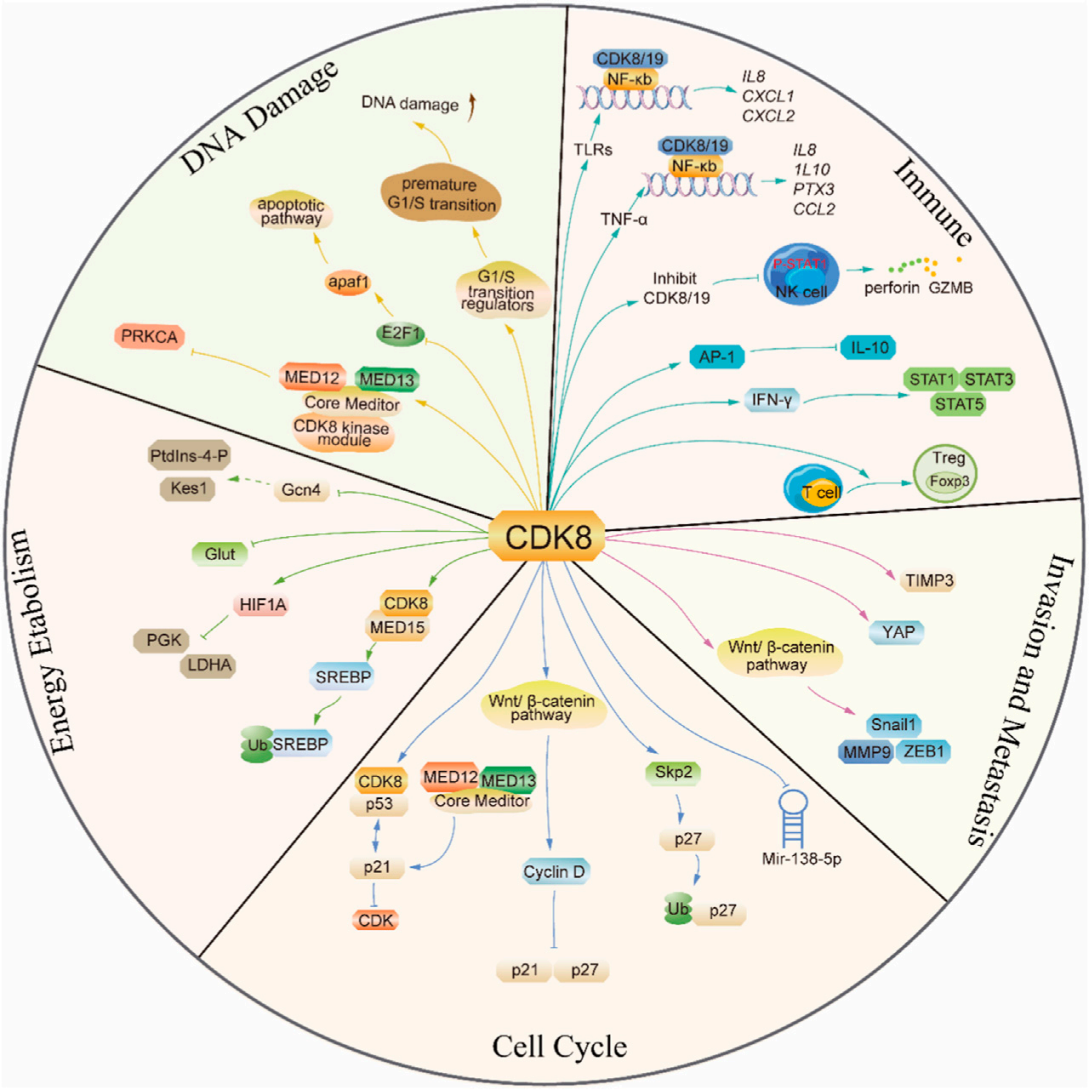
The mechanism diagram of the emerging role of CDK8 in tumorigenesis.

In addition, CDK8 was also found to regulate the cell cycle and promote cellular proliferation in other tumors. Overexpression of CDK8 in Non-small cell carcinoma (NSCLC) partially reverses miR-138-5p-induced G0/G1 phase arrest in NSCLC, thereby promoting the growth of non-small cell carcinoma (Xing et al., 2019). In prostate cancer, miR-372 negatively regulates CDK8 and induces G0/G1 blockade, impeding the proliferation of cancer cells (Kong et al., 2016). Cancer cells are prevented from proliferating when T-474 or T-418 (two structurally differentiated CDK8/19 enzyme inhibitors) are used to treat prostate cancer that is susceptible to these drugs. The number of cells in the G1 phase declines, and the number of cells in the S phase increases as a result of modifications at the mRNA expression level to G1/S transition regulators, causing an early G1/S transition. However, the premature G1/S transition causes DNA damage that ultimately results in ATR-dependent cell death (Nakamura et al., 2018). In addition, CDK8 is associated with G2/M transformation, and loss of CDK8 significantly reduces G2/M blockade-mediated proliferation in metastatic melanoma cell lines (Kapoor et al., 2010) (Figure 3).

### 3.2 CDK8 promotes cancer invasion and metastasis

Tumor cells proliferate endlessly in the primary site, and the formation of an obvious primary tumor lesion often takes several years. Although primary tumors are extremely dangerous, they eventually cause death in only about 10% of patients, and approximately 90% of patients with such tumors eventually die from the growth of a metastatic tumor outside the primary site, suggesting that invasion and metastases occurring at the late tumor stage have a considerable impact on the survival rate of patients and warrant more attention.

CDK8 is amplified in colon cancer, pancreatic cancer, and other cancer (Firestein et al., 2008; Offermann et al., 2022). The proliferation of colon cancer cells that overexpress CDK8 can be effectively inhibited by knocking down CDK8, as reported in previous studies. However, both the knockdown of CDK8 and the inhibition of its kinase activity significantly suppress metastatic growth specifically within the liver. Interestingly, the growth of cecal, splenic, or subcutaneous metastatic colon cancer remained unaffected upon knockdown or inhibition of CDK8 (Liang et al., 2018). From a mechanistic perspective, the inhibition of CDK8 resulted in significant upregulation of TIMP3 gene expression with a concurrent downregulation in the expression of MMPs. The intricate interplay between MMPs and TIMPs, which are natural inhibitors of MMP activity, significantly contributes to the advancement of tumor progression. From a mechanistic perspective, the inhibition of CDK8 resulted in downregulation of matrix metalloproteinase (MMP) and upregulation of tissue inhibitor of metalloproteinases (TIMP) families, specifically TIMP3. The intricate interplay between MMPs and TIMPs, significantly contributes to the advancement of tumor progression. TIMP3 functions as an inhibitor of invasion and metastasis in various types of cancer. MMPs have crucial involvement in encompassing growth factor receptor signaling, angiogenesis, cell adhesion, and apoptosis apart from matrix degradation. On one hand, CDK8 inhibition induces TIMP3 via SMAD-regulated upregulation of miR-181b, which constitutes the primary mechanism underlying the impact of CDK8 on the growth of liver metastases in colon cancer. On the other hand, CDK8 facilitates the upregulation of MMP9 expression via Wnt/β-catenin-mediated transcriptional activation. Altogether, these data suggest that the site-specific tumor inhibitory effect of CDK8 knockdown or inhibition may be related to the environment-specific roles of TIMP3 and MMPs (Liang et al., 2018).

The NOTCH intracellular domain (NICD) plays a pivotal role in tumor differentiation and metastasis by regulating various upstream or downstream factors associated with tumorigenesis, such as MYC or P53, while also promoting angiogenesis and tumor invasion by regulating cell cycle progression. The activation of NOTCH has been proven to possess oncogenic properties in diverse types of cancers, such as T-ALL, breast cancer, lung adenocarcinoma, hepatocellular carcinoma, ovarian cancer, and colorectal cancer (Zhou et al., 2022). CDK8 phosphorylates the serine of the PEST region of NICD1 and subsequently recognizes and ubiquitinates phosphorylated NICD1 through the E3 ligase FBXW7, prompting the ubiquitination of NICD1 for degradation by the proteasome (Fryer et al., 2004). In non-small cell lung cancer, CDK8/FBXW7-mediated NICD1 degradation can be competitively abolished by RFC4 (a DNA replication factor), which results in increased NICD1 stability and promotes NSCLC invasiveness. The competitiveness of RFC4 stems from its approximately 5-fold higher binding affinity to NICD1 compared to CDK8, and the augmented expression level of RFC4 in NSCLC tissues further amplifies its competitive binding to NICD1 over CDK8. In addition, the inhibition of CDK8 can greatly augment the interaction between NICD1 and RFC4, thereby facilitating the invasion of NSCLC (Liu et al., 2021). The activation of YAP is facilitated by CDK8 through a dual mechanism that involves both S127 phosphorylation and direct phosphorylation at T119/S128/S289/S367 via its kinase activity. The CDK8-mediated direct phosphorylation further enhances YAP activation, thereby promoting colon cancer migration. Of note, the expression of CDK8 can be regulated by the cell-adhesion protein Zyxin, a constituent of the focal adhesion complex which is involved in cellular motility, thereby promoting colon cancer cell proliferation (Serrao et al., 2018; Zhou et al., 2018). CDK8, which can be promoted by mutant K-ras by promoting HIF1α expression, acts with mutant K-ras to inhibit GSK-3β and Axin2 expression, thus increasing T-β-catenin and N-β-catenin expression and activating the Wnt pathway. Hence, it increases the expression of Snail1 and ZEB1 and stimulates epithelial–mesenchymal transition in pancreatic cancer (Xu W. et al., 2015). The downregulation of CDK8 in Lung squamous cell carcinoma (LSCC) resulted in the inhibition of β-catenin expression and the promotion of E-cadherin expression, which attenuates FaDu cell invasion. In clinical settings, the expression levels of CDK8 exhibited a positive correlation with both lymph node metastasis and clinical stage (Li et al., 2017) (Figure 3).

### 3.3 CDK8 in energy metabolism

As cancer cells grow and reproduce rapidly, they often consume far more oxygen and nutrients than the blood can supply, which leads to a severe deficiency in oxygen and other nutrients such as glucose in cancer cells. To better adapt to the hypoxic environment, cancer cells must undergo alterations in energy metabolism to enhance ATP production through glycolysis. This metabolic reprogramming results in an increased rate of glucose consumption and glycolysis in cancer cells compared to normal cells (Ganapathy-Kanniappan and Geschwind, 2013).

In colorectal cancer cells, impairing glucose transporter expression, glucose uptake, and glycolytic capacity in both normoxia and hypoxia can be achieved by using the CDK8/CDK19 inhibitor Senexin A or through the knockdown of CDK8. This sensitizes cells to the 2-deoxy-D-glucose (glycolysis inhibitor) (Galbraith et al., 2017). In summary, combining a CDK8 inhibitor with a glycolytic inhibitor holds promise as an approach for treating tumors with high rates of glycolysis.

Numerous studies indicate that HIF1α, which controls the transcription of many glycolytic enzymes including PGK and LDHA, acts as a master transcriptional regulator of the hypoxic response (Majmundar et al., 2010). Multiomics analysis revealed a prompt transcriptional response to acute hypoxia, which is highly dependent on the kinase activity of HIF1α and its cofactor CDK8. Moreover, this response exhibits remarkable conservation across various cancer types (Andrysik et al., 2021). The Mediator-associated kinase CDK8 is currently recognized as necessary for the stimulation of several HIF1α target genes. During normoxia, most HIF1α-targeted genes carry paused RNA pol II. In response to hypoxia, HIF1α binds to chromatin and recruits CDK8-Mediator, BRD4, and SEC-containing proteins, HIF1α binding to chromatin leads to increased histone acetylation, recruitment of CDK8-Mediator, bromodomain protein BRD4, and SEC-containing P-TEFb, leading to increased phosphorylation of RNA pol II CTD and transcriptional elongation. In the case of knockdown of CDK8, HIF1α is capable of binding chromatin and inducing histone acetylation; however, it fails to recruit core Mediators and SECs and facilitate transcriptional elongation (Galbraith et al., 2013).

Another significant metabolic change associated with cancer is dysregulation of lipid metabolism. The three most common lipids that are the raw materials for energy producers, signaling molecules, and cell membrane biogenesis are fatty acids, cholesterol, and phospholipids. Increased lipid synthesis, storage, and absorption are factors in the development of cancer. A complete investigation has been conducted to understand the involvement and regulatory mechanism of lipid metabolism in the development and advancement of tumors. First, reprogramming of lipid metabolism enables tumor cells to produce more energy, which is advantageous for tumor cells to survive in microenvironment with inadequate nutrition. In addition, signaling molecules produced by reprogramming lipid metabolism can activate signaling pathways linked to tumors and encourage the growth, invasion, and metastasis of tumor cells (Li and Kang, 2017; Snaebjornsson et al., 2020).

CDK8 is a negative regulator of lipid biosynthesis. The SREBP family of transcription factors represents the principal regulators governing the transcription of cholesterogenic and lipogenic genes, and RNA-seq research has demonstrated that CDK8 can inhibit the expression of SREBP-dependent lipogenic genes. Mechanistically, CDK8 affects SREBP homeostasis: CDK8 binds directly with the N-terminus of SREBP while phosphorylating it at conserved threonine residues, which necessitates the synergistic action of MED15. Then, SREBP-1C phosphorylated by CDK8 can undergo further ubiquitination and protein degradation (Zhao et al., 2012; Li et al., 2022). The CDK8 module is required for the attenuation of SL-regulated Gcn4 activity, a major transcription factor controlling amino acid homeostasis, thereby participating in lipid metabolic processes mediated by PtdIns-4-phosphate production (PtdIns-4-P) and sterol-binding protein Kes1, a sterol-regulated antagonist of TGN/endosomal phosphatidylinositol-4-phosphate signal (Mousley et al., 2012). The CDK8 module is required for the attenuation of sphingolipid-regulated Gcn4 activity, a major transcription factor controlling amino acid homeostasis, thereby participating in lipid metabolic processes mediated by PtdIns-4-P and Kes1. In obese women, the CDK8 module may be chronically downregulated by increased insulin or insulin-like growth factor signaling, which elicits dysregulation of the activity of transcription factors regulated by the CDK8 module and thus increases the risk of uterine neoplasia (Li et al., 2019) (Figure 3).

### 3.4 CDK8 in DNA damage

Organisms need to detect DNA damage signals to pause or stop cell cycle progression and activate specific DNA repair processes or apoptotic mechanisms. The process must first engage in a signal transduction pathway known as the DNA damage response, which is mediated by protein phosphorylation. Three reactions, cell senescence, apoptosis, and cell carcinogenesis, are likely to take place after cells lose their capacity to effectively repair DNA damage. ATR functions as an apical replication stress response (RSR) regulator as well as a signaling component of cell cycle checkpoint activation and DNA double-strand break (Jackson and Bartek, 2009; Yan et al., 2014). Therefore, by enhancing replication fork stasis, encouraging chromosome breakage, and inducing cytotoxicity, the clinical application of ATR inhibitors has become an emerging strategy for the treatment of tumors.

Knockdown of cyclin C or CDK8 in FaDu and A549 cells was found to promote cellular resistance to ATR and CHK1 inhibition by restricting the formation of DNA:RNA hybrids during S-phase, thereby reducing transcription-related replication stress. In this way, CDK8/cyclin C can keep cancer cells alive by promoting replication stress response of cancer cells and thus reducing DNA damage (Lloyd et al., 2021). CDK8/19 inhibitors promote ATR-dependent cell death and activate the DNA damage response by modulating the mRNA expression levels of regulators involved in the G1/S phase transition. This leads to an accelerated transition from G1 to S phase, resulting in a decreased population of cells in G1 phase and an increase in the number of cells in S phase. Additionally, the combination of CDK8/19 inhibitor T-474 with topoisomerase inhibitor SN-38 (the active metabolite of irinotecan), etoposide, and doxorubicin significantly potentiated their cytotoxic effects in VCaP cells. This synergistic effect may be attributed to the accumulation of DNA damage induced by this combinatorial treatment (Nakamura et al., 2018). CDK8 acts as a switch of the Mediator complex, and its binding state to Mediator can be regulated by PARP proteins (Pavri et al., 2005). PARP inhibitors can cause DNA damage by activating the ATM/ATR-CHK1/CHK2 signaling pathway. Therefore, the combination of PARP inhibitors and CDK8 inhibitors will also be a new direction for tumor treatment (Li et al., 2023).

Radiotherapy can cause various forms of damage to DNA in cells and activate the p53 and E2F1 pathways to repair damage synergistically or independently or induce apoptosis to kill tumor cells (Udayakumar et al., 2010). In colorectal cancer, CDK8 phosphorylates E2F1 at the S375 site depending on its kinase activity rather than transcriptional activity, thus downregulating E2F1 transcriptional activity without affecting its protein stability. After combined IR treatment, knockdown of CDK8 enhanced the transcriptional activity of E2F1, resulting in a higher level of apaf1, triggering the endogenous apoptotic pathway and thus enhancing the sensitivity of colorectal cancer cells to IR (Chen et al., 2020). In addition, in NSCLC cells, when the CDK8 kinase module is structurally intact, the physical interaction between the modules is weakened due to the silencing of MED13L, which ultimately inhibits the expression of the oncogene PRKCA, resulting in the sensitivity of NSCLC cells to radiotherapy (Zhang et al., 2020) (Figure 3).

### 3.5 CDK8 in cancer immune

T Regulatory cells (Tregs) play a dual role in the immune system, serving both protective and pathological functions. They maintain immune homeostasis, suppressing immune responses in various diseases, including cancer. By inhibiting the function of T effector cells, Tregs weaken tumor-killing effects and promote tumor growth, driving the progression of cancer. Tregs, which exhibit upregulated expression of the transcription factor Forkhead box protein P3 (Foxp3), play a crucial role in suppressing excessive immune responses, including autoimmune diseases, making them central negative regulators of the adaptive immune system (Saleh and Elkord, 2020). Recently, CDK8/19 has been shown to be involved in the differentiation of Treg cells. Inhibiting or knocking out CDK8/19 can induce the expression of Foxp3 in naïve CD4^+^ T cells, naïve CD8^+^ T cells, antigen-activated effector/memory CD4^+^ T cells, and antigen-activated effector/memory CD8^+^ T cells. This induction is associated with STAT5 activation, independent of the action of TGF-β, and is not influenced by inflammatory cytokines. This suggests that under physiological conditions, inhibiting CDK8/19 can induce Foxp3 expression in activated conventional T cells, and pharmacological inhibition could potentially convert antigen-specific effector/memory T cells into Foxp3+ Treg cells (Akamatsu et al., 2019). However, there are also studies reporting that the enhanced differentiation of Treg cells by inhibiting CDK8/19 is due to the inhibition of TGF-β signaling, which is associated with the attenuation of IFN-γ-Stat1 signaling and the enhancement of Smad2/3 phosphorylation. The use of CDK8/19 small molecule inhibitors such as CCT251921 or Senexin A to inhibit the activity of CDK8/19 greatly promotes the differentiation of Treg cells and the expression of Treg characteristic genes *Foxp3*, *CTLA4*, *PD-1*, and *GITR*. In an experimental autoimmune encephalomyelitis (EAE) model, treatment with CCT251921 significantly increased the population of Treg cells and improved autoimmune symptoms (Guo et al., 2019).

Natural Killer (NK) cells are an important type of lymphocyte with unique immune cytotoxic functions. They can rapidly identify and kill cells that are diseased or infected, especially those displaying surface markers associated with tumorigenesis. Additionally, NK cells can enhance the responses of other immune cells and antibodies, thereby coordinating and strengthening the overall immune response (Shimasaki et al., 2020). Inhibiting CDK8/19 can suppress the phosphorylation of STAT1 S727 in NK cells and increase the production of the cell lytic molecule perforin and the granule enzyme B (GZMB). This is associated with enhanced cytotoxicity of NK cells *in vitro*. Functionally, this resulted in enhanced NK-cell–mediated lysis of primary leukemia cells (Witalisz-Siepracka et al., 2018; Hofmann et al., 2020). Clinically, the CDK8/19 inhibitor (BI-1347) can be used in combination with modulators of the adaptive immune system, such as anti-PD-1 antibodies and SMAC mimetic BI-8382, to inhibit the growth of solid tumors. This effect is not related to their activity against cancer cells but rather through enhancing NK cell function (Hofmann et al., 2020).

The nuclear factor-κB (NF-κB) transcription factor family is a major regulator of inflammation and has been implicated in cancer (Hoesel and Schmid, 2013). Recent studies have demonstrated that CDK8/19 regulates NF-kB-mediated gene expression in response to various stimuli. Upon stimulation by tumor necrosis factor-alpha (TNF-α), NF-kB heterodimers and CDK8/19 are recruited together to promoters, leading to the expression of early response genes including *Il8*, *Cxcl2*, and *Cxcl3*. Inhibition of CDK8/19 kinase activity can specifically suppress the expression of certain NF-kB target genes while leaving the basal expression of NF-kB-regulated genes unaffected (Chen et al., 2017). In the context of Toll-like receptor (TLR)-mediated gene expression, CDK8 and CDK19, along with NF-κB and C/EBPβ, have been observed to colocalize at the promoter regions of inflammation-related genes like *Il8*, *Il10*, *Ptx3*, and *Ccl2*, facilitating their transcription in a coordinated manner (Yamamoto et al., 2017).

Interferon-gamma (IFN-γ) is a signaling protein that serves as the first line of nonspecific defense against invading pathogens. It is also the most crucial cytokine in anti-tumor immunity (Kursunel and Esendagli, 2016). CDK8 has been shown to phosphorylate STAT1, STAT3, and STAT5 in response to IFN-γ stimulation. These transcription factors are activated by Janus-activated kinases (JAKs) downstream of the interferon receptor. More than 40% of IFN-γ response genes are positively or negatively regulated by CDK8-mediated STAT1 phosphorylation, thereby establishing CDK8 as a crucial modulator of antiviral responses (Bancerek et al., 2013). Under IFN-γ stimulation, the levels of phosphorylated STAT1 decreased when using the CDK8/19 inhibitors Cpd32 and Cortistatin A, confirming the kinase-dependent role of CDK8/19 in regulating antiviral gene expression (Pelish et al., 2015; Koehler et al., 2016).

Interleukin-10 (IL-10) is an anti-inflammatory cytokine produced by various cells, and it has been implicated in the pathogenesis and development of autoimmune diseases and cancer (Mannino et al., 2015). CDK8 has been identified as a target of BRD6989, a small molecule enhancer of IL-10 secretion, and this ability of BRD6989 to enhance IL-10 requires the intact CDK8 complex. During the process of innate immune activation, CDK8/19 acts as a negative regulator of IL-10 production, and this negative regulatory effect is associated with increased AP-1 (a class of transcriptional activator factors primarily composed of proteins encoded by the proto-oncogenes Jun and Fos) activity. This suggests that using CDK8 inhibitors to upregulate IL-10 may be an effective and tolerable therapeutic approach for inflammatory diseases (Johannessen et al., 2017).

Additionally, tissue co-culture models using different primary cells have shown that inhibition of CDK8/19 modulates the expression of various cytokines such as IL-8, IL-17A, and IL-17F. This indicates that CDK8/19 may play a role in the regulation of inflammatory gene expression *in vivo* (Clarke et al., 2016) (Figure 3).

## 4 The clinical role of CDK8 inhibitors

Given the crucial role of CDK8 in regulating gene transcription and cell signaling, its abnormal activity is associated with cancer. Inhibitors of CDK8/19, by suppressing the activity of CDK8/19, hold the potential to intervene in abnormal gene expression, regulate cell proliferation and survival, thus providing new therapeutic avenues and possibilities for cancer treatment. Chemotherapy increases tumor-induced paracrine effects, ultimately leading to drug resistance and the secretion of various pro-tumor cytokines. This secretory phenotype of tumor cells is partly mediated by the damage-induced cell cycle inhibitor p21 (Chang et al., 2001). Senexin A is a natural product and the earliest CDK8 inhibitor, which not only inhibits p21-stimulated transcription *in vitro* and *in vivo* but also suppresses the production of cytokines from damaged cells and the paracrine activity that damages both tumor and normal cells during chemotherapy. This action reverses the paracrine-related anti-apoptotic effects. Therefore, clinically, Senexin A can be used to inhibit tumor resistance to chemotherapy or radiation (Porter et al., 2012).

In breast cancer, approximately 20% of patients overexpress HER2 and receive HER2-targeted therapies such as trastuzumab, pertuzumab, lapatinib, neratinib, and T-DM1. Despite the effectiveness of these drugs in metastatic HER2-positive breast cancer, around 70% of patients exhibit intrinsic resistance. Senexin B and SNX631 (Selective CDK8/19 inhibitors) have shown synergistic interactions with lapatinib and trastuzumab in a group of HER2^+^ breast cancer cell lines, overcoming and preventing cell resistance to HER2-targeted drugs. This synergy is partly mediated through the PI3K/AKT/mTOR pathway and involves the synergistic inhibition of STAT1 and STAT3 phosphorylation at the S727 site by CDK8/19 and HER2-targeted drugs (Ding et al., 2022).

In breast cancer cells BT474 (ER and HER2 positive) and SKBR3 (ER negative, HER2 positive) exposed long-term to EGFR-targeted small molecules (gefitinib, erlotinib) and colon cancer cells SW48 exposed long-term to anti-EGFR monoclonal antibody cetuximab, the development of resistance was analyzed. In all cases, small subpopulations initially showed growth inhibition with a single dose of the drug, followed by regrowth and development of resistance. However, this adaptation was always prevented by the addition of selective CDK8/19 inhibitors (Senexin B or 15w), even though these inhibitors alone had moderate or no effect on cell growth. These results suggest that combining EGFR-targeted drugs with CDK8/19 inhibitors may delay or prevent the development of resistance to therapy. However, CDK8/19 inhibitors did not enhance the effects of EGFR inhibitors or overcome acquired resistance to these drugs, indicating that the prevention of resistance is likely due to the inhibition of transcriptional reprogramming by CDK8/19 inhibitors (Sharko et al., 2021). Furthermore, Senexin B has become the first selective CDK8/19 inhibitor to enter clinical trials, used in combination with aromatase inhibitors or fulvestrant for advanced ER-positive breast cancer (Zhang et al., 2022).

Immunotherapy has demonstrated significant efficacy in many types of cancer and is considered a key direction for future cancer treatments. The loss of CDK8 in NK cells enhances the anti-tumor response against B16F10 melanoma cells and *v-abl* ^+^ lymphoma cells. In a slowly progressing leukemia model system, CDK8 loss prolongs the disease latency. These results suggest that therapies targeting CDK8 in cancer patients may enhance NK cell responses against tumor cells, providing new avenues for immunotherapy (Witalisz-Siepracka et al., 2018). In other hematologic malignancies, Cortistatin A, a selective CDK8 inhibitor derived from natural products, can reduce the growth of AML with a megakaryocytic phenotype (such as MOLM-14). This may be due to the inhibition of phosphorylation of STAT1 at Ser727 (Nitulescu et al., 2017).

Therefore, combining CDK8 inhibitors with immunotherapy, chemotherapy, or targeted therapy may result in synergistic effects, enhancing treatment outcomes. This combined treatment strategy holds the potential to bring new breakthroughs and possibilities to cancer therapy.

## 5 Conclusion and prospects

As a cyclin-dependent kinase, CDK8 not only controls the cell cycle through interactions with other proteins but also phosphorylates transcription factors and participates in transcription processes, thus controlling a number of signaling pathways linked to tumor formation, including transcriptional regulators, signaling systems, modifications in the tumor cycle, DNA damage repair, energy metabolism, invasion, and metastasis. Due to the significance of CDK8 as a tumor-promoting factor, CDK8 inhibitors have been developed for the treatment of malignancies. The investigation of combining CDK8 inhibitors with chemotherapy or radiotherapy is warranted in future research, as it potentially will enhance the therapeutic efficacy and long-term survival rate of cancer patients.
